# Cholesterol and Cholesterol‐Derived Molecules Differentially Modulate Neuronal Kv7.2/7.3 Channels

**DOI:** 10.1002/ardp.70280

**Published:** 2026-06-15

**Authors:** Elif Karabatak, Ronewa Nematswerani, Vivian Meiritz, Nicole Rychlik, Guiscard Seebohm, Frank Glorius, Thomas Budde

**Affiliations:** ^1^ Institut für Physiologie I Universität Münster Münster Germany; ^2^ Organisch‐Chemisches Institut Universität Münster Münster Germany; ^3^ Klinik für Neurologie Universität Münster Münster Germany; ^4^ Cellular Electrophysiology and Molecular Biology, Institute for Genetics of Heart Diseases (IfGH) Universität Münster Münster Germany

**Keywords:** cholesterol, electrophysiology, ion channels, Kv7, modulators

## Abstract

Kv7 potassium channels generate slowly activating, non‐inactivating outward potassium currents and are critical regulators of cellular excitability. While cholesterol is known to modulate multiple ion channels, its concentration‐dependent effects and the influence of cholesterol‐derived molecules on Kv7 channels remain insufficiently characterized. In this study, we investigated the effects of cholesterol and cholesterol‐derived molecules, including steroid hormones and chemically modified imidazolium‐based cholesterol derivatives (CHIMs) on Kv7.2/7.3 channels heterologously expressed in HEK293FT cells using conventional whole‐cell patch‐clamp recordings. Application of high cholesterol concentrations (1 mM) significantly reduced Kv7.2/7.3 current amplitudes over a wide range of potentials without changing voltage‐dependent current characteristics. CHIMs also reduced currents, whereas a fluorescent derivative, CHIM‐L‐NBD, unexpectedly enhanced Kv7.2/7.3 currents. In contrast, the NBD moiety alone had no effect. Progesterone and 17β‐estradiol inhibition of Kv7.2/7.3 currents was most evident at strongly depolarized potentials. For progesterone this was associated with a change in the slope of the activation curve. These findings demonstrate that Kv7.2/7.3 channels respond differentially to structural modifications of cholesterol and to steroid hormones thereby suggesting that different cholesterol‐derived molecules may serve as tool compounds with potentially opposing modulatory effects on Kv7.2/7.3 channels.

## Graphical abstract

Cholesterol, steroid hormones, and cholesterol‐based imidazolium salts (CHIMs) inhibit Kv7.2/Kv7.3 channels. Notably, NBD‐tagged CHIM‐L enhances currents. These findings demonstrate that both chemical modifications and linker length differentially modulate Kv7 activity, revealing variant‐specific sensitivities and potential pharmacological targets for channel regulation.

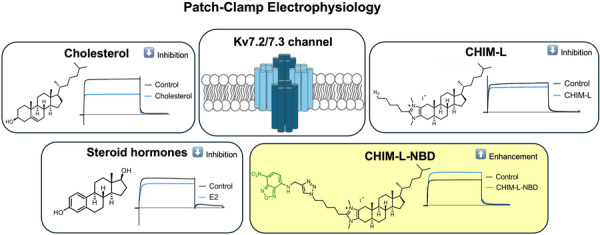

## 1 Introduction

Kv7 potassium channels (encoded by KCNQ genes) are key regulators of membrane excitability in neurons and other excitable cells. The heteromeric Kv7.2/7.3 channel complex underlies the neuronal M‐current, a slowly activating, non‐inactivating potassium conductance that stabilizes the resting membrane potential and limits repetitive firing [1, 2]. Dysfunction of Kv7.2/7.3 channels has been implicated in epilepsy, neuropathic pain, and neurodevelopmental disorders [3, 4, 5].

Despite significant progress in understanding Kv7 channel physiology, the development of selective pharmacological modulators remains a major challenge [5, 6, 7]. Existing compounds, such as retigabine, have proven valuable as tool compounds but lack subtype selectivity and exhibit off‐target effects, limiting their clinical applicability [7, 8, 9]. The identification of new modulators that enable subtype‐specific targeting and improved mechanistic insight therefore remains an important goal. Current strategies include structure‐based drug design and high‐throughput screening approaches, often guided by endogenous modulators such as lipids and steroid hormones as well as by rational designed benzamide‐related compounds [10, 11].

Beyond canonical regulation by phosphatidylinositol‐4,5‐bisphosphate (PIP_2_) [12, 13], membrane lipids have emerged as important modulators of ion channel function [14]. In particular, cholesterol can influence channel activity both indirectly, by altering biophysical properties of the cell membrane, and directly, through specific lipid‐protein interactions [14, 15, 16]. However, its effect on Kv7 channels remain incompletely understood, and available data are limited and partly contradictory [15, 17]. Structurally related steroids, including 17β‐estradiol and progesterone, have likewise been shown to modulate ion channel activity, further supporting a role for sterol‐based molecules in channel regulation [18, 19].

To address experimental limitations associated with native cholesterol, chemically modified cholesterol analogs have been developed. Cholesterol‐based imidazolium salts (CHIMs) represent functional cholesterol mimetics that retain the rigid sterol scaffold while introducing a permanently charged headgroup [20, 21]. This modification improves aqueous dispersibility, facilitates controlled cellular delivery, and enables chemical functionalization without abolishing membrane association. Originally developed as tools to study cholesterol dynamics in biological membranes, CHIMs provide a versatile platform to probe cholesterol‐sensitive processes while minimizing nonspecific aggregation effects [22]. However, their impact on ion channel function, including Kv7.2/7.3 channels, has not yet been systematically investigated.

Importantly, the functional outcome of Kv7 channel modulation appears to be sensitive to the molecular structure. Subtle chemical modifications of channel modulators can lead to pronounced changes in channel behavior, including inversion of modulatory effects from activation to inhibition [23]. While the compounds investigated in the present study differ substantially in their physicochemical properties and likely modes of interaction from classical small‐molecule Kv7 modulators, this general principle may nevertheless be relevant when interpreting our findings. In particular, structurally related cholesterol‐derived molecules examined in this study produce distinct and, in part, opposing effects on Kv7.2/7.3 channel activity. Interestingly, recent work has demonstrated that subtype‐selective Kv7.2 modulation can be achieved by targeting specific residues within defined binding pockets [11]. Although binding‐site‐specific mechanisms were not addressed in the present study, achieving subtype specificity by ligand discovery and optimization further underscores the importance of structural determinants in Kv7 channel modulation and supports the concept of rational design of channel modulators.

Building on these considerations, we investigated how cholesterol, steroid hormones, and CHIMs modulate Kv7.2/7.3 channel activity, focusing on whether structural modifications of the cholesterol scaffold influence channel regulation. Our experimental recording approach using manual whole‐cell patch‐clamp aimed to prove the feasibility of a low‐throughput approach using rapid substance application and effective compound concentrations as an initial screening approach.

## 2 Results and Discussion

### 2.1 High Cholesterol Concentrations Reduce Kv7.2/7.3 Currents

The activity of ion channels is finely regulated by membrane cholesterol, the most abundant lipid component of the plasma membrane of mammalian cells. However, the mechanisms underlying cholesterol modulation, and the effects of other steroids, remain largely unexplored, and data specifically addressing Kv7.2/7.3 channels are limited.

**Figure 1:**
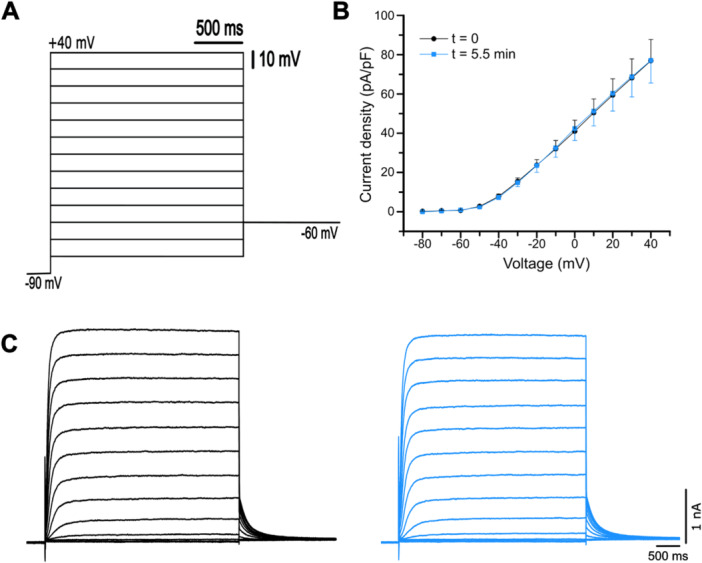
Control measurements for currents generated by Kv7.2/Kv7.3 channels over time. (A) Voltage protocol used to elicit membrane outward currents. (B) Current density‐voltage relationships for currents recorded at the end of 2 s depolarizing pulses at two time points under control conditions. Data are shown as mean ± SEM (*n* = 8; independent cells). (C) Representative current traces obtained under control conditions, elicited by voltage steps ranging from −80 mV to +40 mV at the beginning of the whole cell recording (black traces) and 5.5 min later (blue traces).

First, we addressed the functional relevance and stability of our new Kv7.2/7.3 channel construct expressed with a fixed 1:1 stoichiometry in transiently transfected HEK293FT cells using a KCNQ3‐P2A‐KCNQ2‐DsRED plasmid. Depolarizing step protocols (Figure 1A) evoked membrane currents with kinetic and voltage‐dependent properties characteristic of an M‐type potassium current with a threshold potential of about −60 mV (Figure 1B). Depolarizing voltage commands induced slowly activating outward currents that showed no time‐dependent inactivation and fast deactivating tail components (Figure 1C). The observed current was therefore in accordance with the typical voltage range of M‐currents [2, 24]. When the stability of the evoked current was assessed, maximal current densities ranged from 41.8 to 122.7 pA/pF, with a mean of 76.9 pA/pF ± 10.8 pA/pF, which was unchanged over the observed time period of 5.5 min (77.1 pA/pF ± 11.5 pA/pF; *n* = 8; Figure 1B,C). These data indicate that the heterologous expression system established here enables targeted overexpression of Kv7.2/7.3 channels, resulting in currents that are stable over time with minimal contributions from endogenous channels, thereby allowing the investigation of time‐dependent compound effects on the channel of interest.

In the following, we investigated the effect of cholesterol and cholesterol‐derived molecules on Kv7.2/7.3 channels using a bath perfusion system allowing to switch between different solutions in less than a second. Therefore, the recorded cell was placed in the laminar flow of the application pipette. Following establishment of the conventional whole‐cell configuration and an equilibrium phase of 2–3 min, outward currents were recorded under control conditions in a solution containing the respective solvent of the tested compound. Solvents alone had no effect on Kv7.2/7.3 currents. Cells were then perfused with a compound‐containing solution for 2 min prior to subsequent recordings.

**Figure 2:**
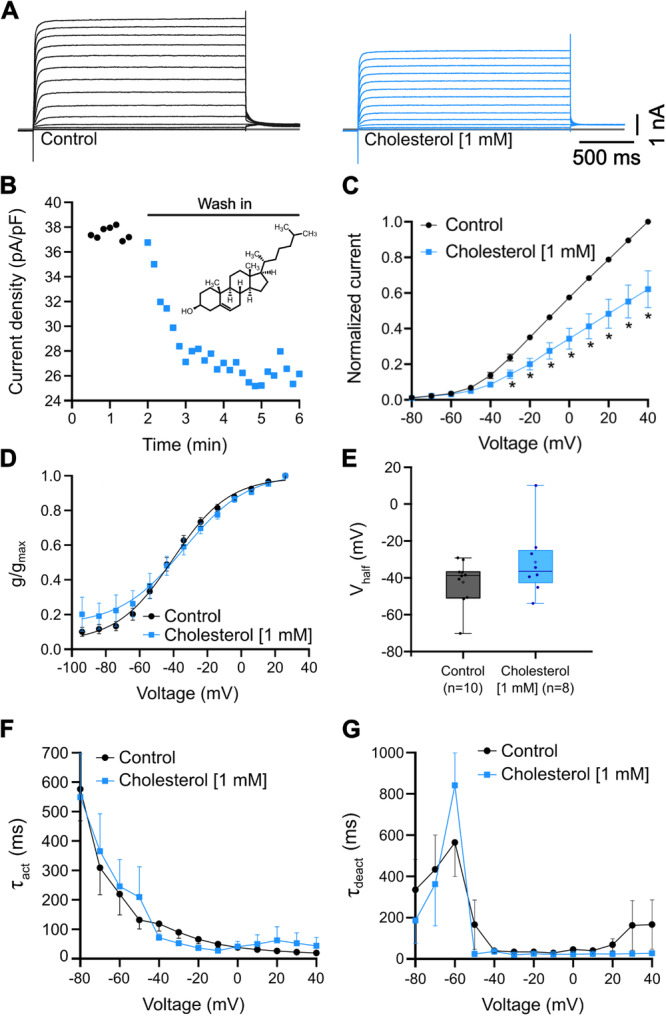
Effects of cholesterol on Kv7.2/7.3 channel currents in HEK293FT cells. (A) Representative whole‐cell patch‐clamp recordings before (black) and after 2 min perfusion with 1 mM cholesterol (blue). (B) Exemplary time course of the Kv7.2/Kv7.3 current amplitude following wash in of 10 µM cholesterol. Outward currents were elicited by depolarizing steps to +20 mV at 10 s intervals. The chemical structure of cholesterol is shown. (C) Mean of normalized step currents (*n* = 10; independent cells); *p < 0.05, paired *t*‐test. Data presented as mean ± SEM; cholesterol reduced Kv7.2/7.3 current amplitudes at potentials ≥ −30 mV. (D) Activation curves obtained from calculating conductance before (black symbols) and after application of 1 mM cholesterol (blue symbols). The smooth lines were plotted by fitting a Boltzmann distribution to the data points. (E) Boxplots of the V_half_ values obtained from Boltzmann fits of currents recorded before (black symbols) and after application of 1 mM cholesterol (blue symbols). The box displays the 25%–75% quartile, the whiskers are the minimum and maximum values, the median is displayed by a straight line and the mean by an asterisk. (F and G) Time constant values of activation (F; τ_act1_ is shown) and deactivation (G; τ_deact1_ is shown) kinetics as a function of step potential before (black symbols) and after cholesterol (1 mM) application (blue symbols).

First cholesterol was tested (Figure 2A). Increasing cholesterol concentrations of 10 µM (in 0.1% DMSO or CHCl_3_; 9 of 15 cells revealed a decrease independent of solvent), 100 µM (in 0.1% CHCl_3_; 5 of 7 cells revealed a decrease) and 1 mM (in 0.1% CHCl_3_; 10 of 10 cells revealed a decrease) resulted in a nominally dose‐dependent current decrease of −7.6% ± 3.7% (*n* = 15; *p* > 0.05), −8.4% ± 2.8% (*n* = 7; *p* > 0.05) and −37.6% ± 10.4% (*n* = 10; *p* < 0.05) at +40 mV, respectively. In our hands, the application of cholesterol (example with 10 µM) resulted in an inhibition of Kv7.2/Kv7.3 currents that was evident at ~30 s (Figure 2B), and then reached a plateau within ~2 min [24]. These finding points to effective substance impact also at a low concentration. No changes in membrane capacitance were found in absence (42.1 ± 6.0 pF) and presence of cholesterol (42.0 ± 6.2 pF; *n* = 24) for cells revealing a current reduction and all tested concentrations. Due to the significant current reduction at 1 mM, we next explored whether cholesterol at that concentration affected the electrophysiological characteristics of Kv7.2/Kv7.3 channels (Figure 2C−G). A significant decrease in outward potassium current was observed at potentials ≥ −30 mV (Figure 2C). Quantification of normalized step currents at +30 mV revealed a significant reduction in the presence of cholesterol (relative values: 0.55 ± 0.09; absolute values: 114.9 ± 42.1 pA/pF) compared with control conditions (relative values: 0.90 ± 0.01; absolute values: 166.9 ± 40.7 pA/pF; *n* = 10; *p* < 0.05). No significant shift in the voltage‐dependent activation curve was found before (control: V_half_ = −42.4 ± 3.9 mV; *k* = 17.4 ± 2.2 mV) and after wash in of cholesterol (V_half_ = −31.5 ± 6.9 mV; *k* = 17.4 ± 1.5 mV; *n* = 8; Figure 2D,E). Next, we characterized the effect of cholesterol on the voltage‐dependent kinetics of the macroscopic Kv7.2/7.3 currents. A two‐exponential function was fitted either to activation (τ_act_) or deactivation (τ_deact_) current waveforms. Values for τ_act1_ strongly decreased from 576.4 ± 107.8 ms at −80 mV to 19.5 ± 3.5 ms at +40 mV under control conditions (*n* = 10; Figure 2F). In the presence of cholesterol, τ_act1_ decreased from 549.3 ± 147.9 ms at −80 mV to 43.5 ± 28.8 ms at +40 mV (*n* = 9; Figure 2F). Values for τ_act2_ ranged from 111.2 ± 99.3 s at −80 mV to 476.4 ± 160.2 s at +40 mV under control conditions (*n* = 10; data not shown) and from 401.6 *±* 134.8 s at −80 mV to 156.6 ± 114.2 s at +40 mV in the presence of cholesterol (*n* = 9; data not shown). In the same voltage range τ_deact1_ varied between 335.1 ± 147.3 ms (−80 mV) and 166.4 ± 119.3 ms (+ 40 mV), revealing a maximum of 564.1 ± 165.4 ms at −60 mV (*n* = 8; Figure 2G) under control conditions. In the presence of cholesterol τ_deact1_ varied between 186.0 ± 111.91 ms (−80 mV) and 26.9 ± 8.3 ms (+ 40 mV), revealing a maximum of 841.2 ± 157.6 ms at −60 mV (*n* = 6; Figure 2G). Values for τ_deact2_ varied between τ_deact2_ = 21.82 ± 10.35 s at −80 mV and τ_deact2_ = 14.2 ± 8.1 s at +40 mV under control conditions (*n* = 8). In the presence of 1 mM cholesterol values varied between τ_deact2_ = 12.5 ± 8.7 s at −80 mV and τ_deact2_ = 269.27 ± 125.49 ms at +40 mV (*n* = 6; data not shown). No significant differences between both conditions were found for current kinetics.

These results indicate that cholesterol very quickly affects Kv7.2/7.3 currents presumably by entering the plasma membrane. However, rather strong increases in cholesterol levels are necessary to exert a robust inhibitory effect on Kv7.2/7.3 currents. This reduction is not based on changes in the activation curve or current kinetics. Therefore, we focused on analyzing the relative current‐voltage relationships in the following.

### 2.2 Evaluation of Cholesterol‐Derived Molecules on Kv7 Channel Activity

**Figure 3:**
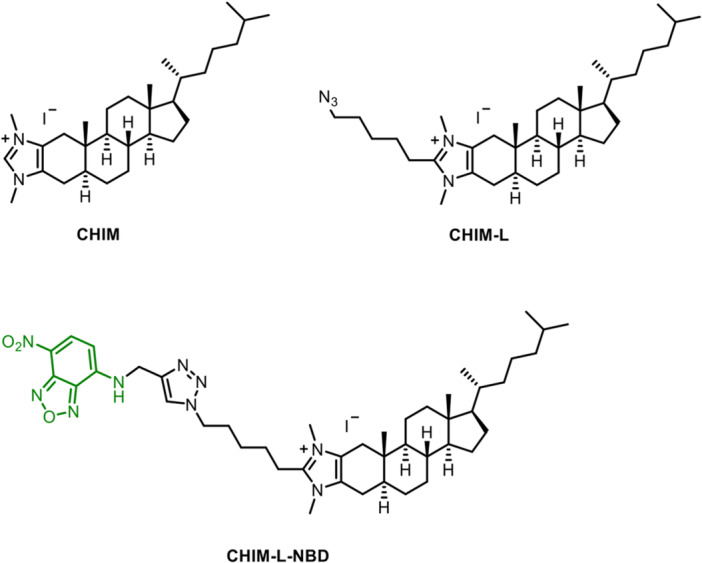
Chemical structures of cholesterol‐based imidazolium salts (CHIMs) and derivatives tested on Kv7.2/7.3 channels. Structures include CHIM, CHIM‐L, and CHIM‐L‐NBD; synthesized following Rakers et al. (2018) [20] and Zheng et al. (2023) [21].

In the following, cholesterol‐derived molecules amenable to chemical modification were evaluated for their effects on Kv7.2/7.3 channel activity. Previously described cholesterol‐based imidazolium salts (CHIMs) [16] developed to probe cholesterol dynamics in cells (Figure 3) were evaluated. In this context, the experimental design was chosen to enable direct comparison of structurally related compounds under standardized conditions, focusing on relative changes in current amplitude as a robust and comparable functional readout.

#### 2.2.1 CHIM and CHIM‐L Reduce the Kv7.2/7.3 Current

Following control recordings (with 0.1% DMSO), CHIM compounds were applied at 10 µM (in 0.1% DMSO) for 2 min prior to the current recordings. As has been observed for cholesterol, at low concentrations CHIM compounds reduced current amplitudes not all recorded cells.

**Figure 4:**
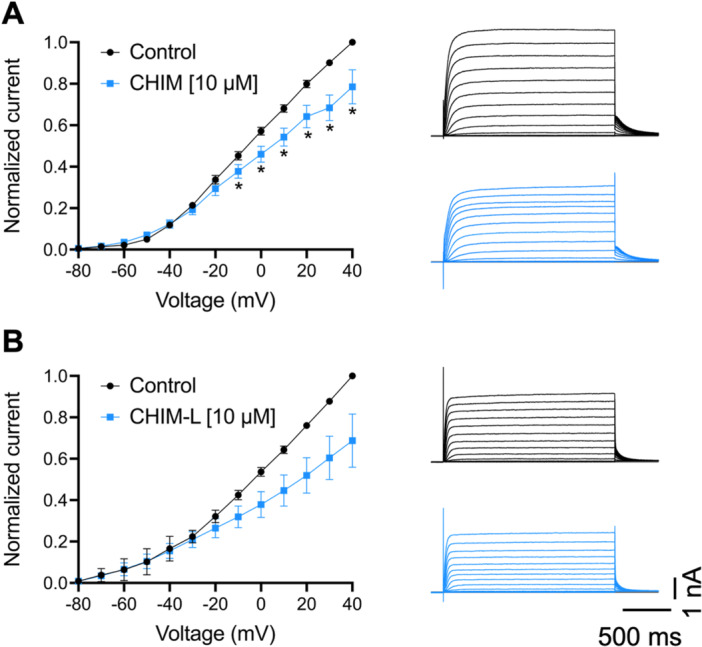
Modulation of Kv7.2/7.3 currents by CHIMs. (A and B) Whole‐cell patch‐clamp recordings of Kv7.2/7.3 channels in HEK293FT cells. Current‐voltage relationships of normalized currents under control conditions and in the presence of (A) CHIM (*n* = 8; independent cells) or (B) CHIM‐L (*n* = 3; independent cells). Currents were evoked by depolarizing steps from −80 to +40 mV. Representative current traces before (black) and after compound application (blue) are shown. Data are shown as mean ± SEM; *p < 0.05, paired *t*‐test.

First, CHIM was tested. The current‐voltage relationship revealed significantly (*p* < 0.05) reduced normalized currents at potentials ≥ −10 mV. Normalized step currents at +30 mV decreased from 0.90 ± 0.01 under control conditions to 0.69 ± 0.06 following CHIM application (*n* = 8; Figure 4A). Absolute current densities at +30 mV were 70.7** ±±** 20.9 pA/pF and 87.4 ± 23.1 pA/pF in the presence and absence of CHIM, respectively.

Next, to assess whether linker modification affects activity, the linker‐containing analogue CHIM‐L was tested under the same conditions as described above. At 10 µM, CHIM‐L compromised membrane integrity, allowing stable recordings in only three cells and resulting in a non‐significant reduction in current amplitudes (normalized current at +30 mV: control 0.88 ± 0.01; vs. CHIM‐L 0.60 ± 0.10; *n* = 3; *p* = 0.12; Figure 4B). Absolute current densities at +30 mV were 55.2 ± 32.1 pA/pF and 110.5 ± 74.0 pA/pF in the presence and absence of CHIM‐L, respectively. Despite these limitations, the observed trend is consistent with the effects of related compounds and therefore supports the overall pattern of inhibitory modulation described in this study. Reducing the concentration of CHIM‐L to 1 µM preserved membrane integrity but produced no detectable effect on Kv7.2/7.3 currents (*n* = 8; data not shown).

#### 2.2.2 CHIM‐L‐NBD Enhances the Kv7.2/7.3 Current

**Figure 5:**
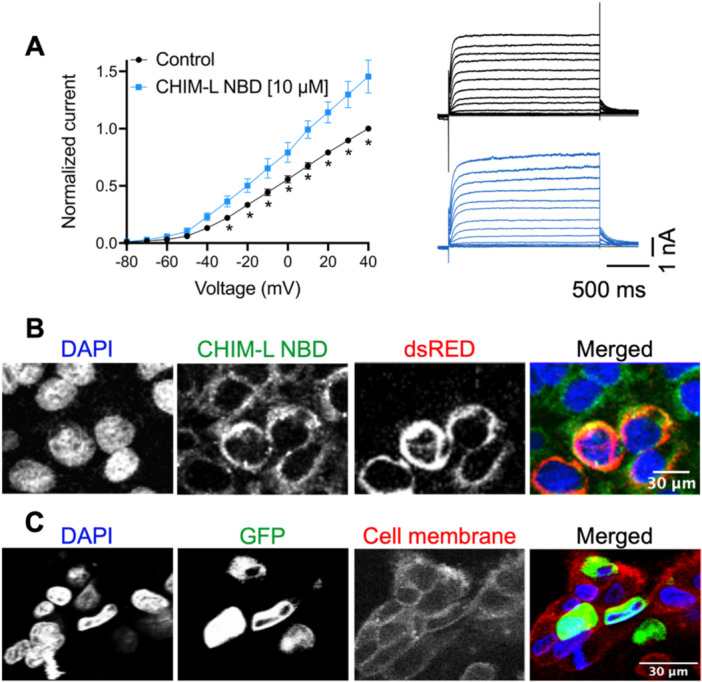
Modulation of Kv7.2/7.3 currents by CHIM‐L‐NBD. (A) Whole‐cell patch‐clamp recordings of Kv7.2/7.3 channels in HEK293FT cells. Current–voltage relationships of normalized currents under control conditions and in the presence of 10 µM CHIM‐L‐NBD. Currents were evoked by voltage steps ranging from −80 to +40 mV in 10 mV increments. Representative current traces before (black) and after compound application (blue) are shown. Data are displayed as mean ± SEM; *p < 0.05, paired *t*‐test (*n* = 7; independent cells). (B) Confocal fluorescence images of transfected HEK293FT cells incubated with 10 µM CHIM‐L‐NBD. DAPI (blue): nuclei; CHIM‐L‐NBD (green); DsRED‐tagged Kv7.2/7.3 channels (red). Scalebar indicates 30 µm and is valid for all images. (C) Confocal fluorescence images of transfected HEK293FT cells stained for cell membrane. DAPI (blue): nuclei; GFP‐tagged Kv7.2/7.3 channels (green); cell membrane (red). Scalebar indicates 30 µm and is valid for all images.

In order to enable multimodal analysis, a functionalized CHIM‐L‐derivative allowing fluorescent compound detection was tested. Unexpectedly, conjugation of CHIM‐L to the NBD fluorophore (CHIM‐L‐NBD) increased Kv7.2/7.3 currents at +40 mV (Figure 5A) in a roughly dose‐dependent manner when 1 µM (9.1% ± 4.2%, *n* = 8; *p* > 0.05), 5 µM (12.4% ± 5.5%, *n* = 9; *p* > 0.05), 10 µM (41.0% ± 12.5%, *n* = 7; *p* < 0.05) and 20 µM (27.9% ± 18.3%, *n* = 9; *p* > 0.05) concentrations were tested. The current‐voltage relationship revealed significantly enhanced normalized currents at potentials ≥ −30 mV when 10 µM CHIM‐L‐NBD was applied. At +30 mV normalized (control: 0.79 ± 0.03; CHIM‐L‐NBD: 1.23 ± 0.11, *n* = 7; *p* < 0.05; Figure 5A) and absolute currents (control: 86.7 ± 23.4 pA/pF; CHIM‐L‐NBD: 118.7 ± 31.9; *n* = 7; *p* < 0.05) were significantly increased. NBD alkyne alone (10 µM) did not alter currents over the investigated voltage range. At +30 mV relative (control: 0.90 ± 0.01; NBD: 0.87 ± 0.04) and absolute (control: 75.8 ± 10.3 pA/pF; NBD: 73.3 ± 10.1; *n* = 10; *p* > 0.05; data not shown) currents were unchanged, indicating that potentiation depends on the CHIM‐L scaffold.

In order to take advantage of the fluorescent property of CHIM‐L‐NBD, confocal microscopy was used. Confocal images indicated membrane localization of CHIM‐L‐NBD and DsRED‐tagged Kv7.2/7.3 channels (Figure 5B), confirming target accessibility in transfected cells and successful functionalization of the compound.

As the increase in Kv7 currents induced by CHIM‐L‐NBD was unexpected, an independent validation was performed using an alternative GFP‐tagged construct (KCNQ3‐P2A‐KCNQ2‐GFP). Recordings under otherwise identical experimental conditions confirmed that CHIM‐L‐NBD significantly (*p* < 0.05) increased Kv7.2/7.3 currents at potentials between −40 to −10 mV (data not shown) and confocal microscopy confirmed membrane localization of GFP‐tagged Kv7.2/7.3 channels (Figure 5C). It is interesting to note that the membrane stain (Deep Red) and CHIM‐L‐NBD revealed robust and very similar staining of the plasma membrane.

#### 2.2.3 Implications for Cholesterol Modulation

Our findings reveal compound‐specific modulation of Kv7 channels by cholesterol‐derived molecules: CHIMs and CHIM‐L trend toward inhibition but show limited stability or reduced efficacy under these conditions, whereas CHIM‐L‐NBD shows activation (in two independent experimental approaches), indicating reproducibility in experimental conditions using expression systems. The lack of NBD effect alone suggests synergistic interplay between the cholesterol scaffold, linker, and fluorophore, potentially via altered membrane cholesterol dynamics or direct channel interactions, warranting further structural optimization for selective Kv7 activators.

The concentration required to observe effects, particularly for cholesterol, may exceed physiological levels but our results are very similar to published observations. First, current reduction occurs within 10th of milliseconds and the results of our imaging experiments confirm the fast loading of cholesterol‐derived compounds into the membrane within a few minutes. Very similar observations have been made before for Kv7.2/7.3 and TRPC3 channels [24, 25] and are compatible with the finding that free cholesterol transfer from lipoprotein particles to the plasma membrane occurs immediately upon contact and is driven by a concentration gradient [26]. We observed Kv7.2/7.3 current reduction by cholesterol at all concentrations with the percentage of responding cells increasing with the concentration. No changes in membrane capacitance were observed, thereby excluding a reduced current density based on increased membrane area. The lack of significant effects at lower cholesterol concentrations may reflect limited effective membrane incorporation under aqueous conditions, which could contribute to the relatively high concentrations required to observe significant electrophysiological effects. However, under acute application conditions in a heterologous expression system with short application times, higher concentrations are often necessary to ensure sufficient membrane partitioning of highly lipophilic compounds. Therefore, the present results should be interpreted as general pharmacological rather than specific physiological modulation. And second, it has been shown before that cholesterol enrichment by a methyl‐β‐cyclodextrin/cholesterol complex inhibited Kv7.2/7.3 currents but did not change their voltage‐dependent steady state activation and current kinetics [24]. The inhibition of Kv7.2/7.3 channels by membrane cholesterol enrichment drive either direct cholesterol‐channel interaction or changes in the physical properties of the plasma membrane. Chemically modified cholesterol analogs may help to distinguish these possibilities.

We therefore tested a first set of CHIM compounds. Two of them (CHIM, CHIM‐L) revealed effects consistent with cholesterol with respect to direction and magnitude of current modulation, but using lower concentrations. Based on the assumption that an optimum cholesterol level in the plasma membrane is required for the proper functioning of Kv7.2/Kv7.3 channels and that cultured HEK293 cells offer these conditions, deviations from this optimum reduce Kv7.2/7.3 current [24]. Since natural cholesterol is assumed to have plasma membrane concentrations typically ranging from 10 to 30 mol% [27], it seems that high levels of endogenously applied cholesterol (hundreds of µM to mM) are necessary to achieve robust modulation of ion channels that occurs within tens of milliseconds [24, 25]. For cholesterol‐derived compounds with altered molecule structure, like CHIM and CHIM‐L, lower concentrations (10 µM) are needed to achieve current inhibition.

Surprisingly, addition of the functional NBD group to CHIM‐L converted the inhibitory effect into channel activation in the present study. It has been observed before that compounds sharing the same chemical scaffold but differing in tail modifications and structural dimensions have opposite functional effects on Kv7.2 channels [23]. Future detailed biophysical characterization of Kv7.2/Kv7.3 channels, together with further modifications of the CHIM‐L structure, will be required to resolve the underlying mechanisms.

### 2.3 Effects of Steroid Hormones on Kv7.2/7.3 Current

#### 2.3.1 Steroid Hormones Reduce Kv7.2/7.3 Current

**Figure 6:**
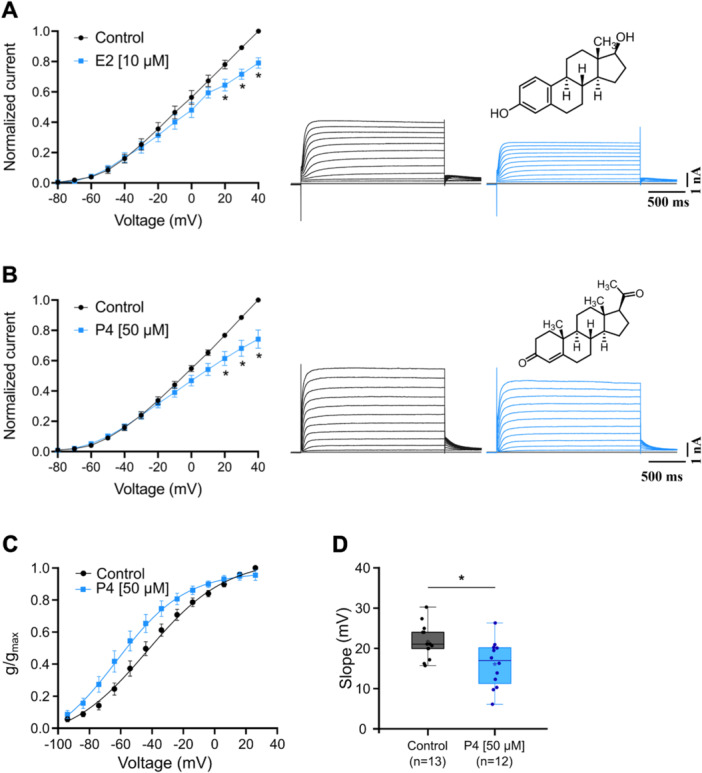
Modulation of Kv7.2/7.3 currents by steroid hormones. (A and B) Whole‐cell patch‐clamp recordings of Kv7.2/7.3 channels in HEK293FT cells. Current–voltage relationships of normalized currents under control conditions and in the presence of (A) 17β‐estradiol (E2; *n* = 8; independent cells) and (B) progesterone (P4; *n* = 13; independent cells) are shown. Currents were evoked by depolarizing steps from −80 to +40 mV. Representative current traces before (black) and after compound application (blue) are shown. Data are shown as mean ± SEM; *p < 0.05, paired *t*‐test. Compound structures are shown above the traces. (C) Activation curves obtained from calculating conductance before (black symbols) and after application of 50 µM P4 (blue symbols). The smooth lines were plotted by fitting a Boltzmann distribution to the data points. (D) Boxplot of the slope values obtained from Boltzmann fits of currents recorded before (black symbols) and after application of 50 µM progesterone (blue symbols). The box displays the 25%–75% quartile, the whiskers are the minimum and maximum values, the median is displayed by a straight line, and the mean by an asterisk.

Following control recordings (with 0.01% EtOH), application of 17β‐estradiol (E2, 10 µM, 2 min, in 0.01% EtOH) significantly reduced Kv7.2/7.3 currents (Figure 6A). At +30 mV, normalized (control: 0.88 ± 0.01; E2: 0.75 ± 0.03) and absolute current values (control: 66.0 ± 15.6 pA/pF; E2: 57.5 ± 14.7 pA/pF) decreased significantly (*n* = 8; *p* < 0.05). A peculiar kink in the current‐voltage relationship at depolarized potentials** >** +10 mV may point to a voltage‐dependent effect. However, when activation curves were analyzed, no significant differences between control conditions (V_half_ = −46.4 ± 9.6 mV; *k* **=** 18.6 ± 1.2 mV; *n* **=** 8) and application of E2 (V_half_ = −61.5 ± 3.4 mV; *k* = 16.6 ± 2.3 mV; *p* > 0.05; data not shown) were found.

Next, progesterone (P4) was tested. Following control recordings (with 0.01% EtOH), application of 10 µM P4 (2 min, in 0.01% EtOH) revealed no change in Kv7.2/7.3 currents. At a higher concentration (Figure 6B), 50 µM P4 significantly reduced Kv7.2/7.3 currents at depolarized potentials more positive than +10 mV. At +30 mV, normalized (control: 0.89 ± 0.01; P4: 0.75 ± 0.03) and absolute (control: 81.1 ± 14.8 pA/pF; P4: 61.8 ± 9.7 pA/pF) current values were significantly reduced (*n* = 13; *p* < 0.05). As observed with E2, the inhibitory effect of P4 appeared at depolarized potentials (> +10 mV) associated with a flattening of the current‐voltage relationship. Therefore, activation curves were analyzed (Figure 6C). While no significant (*p* > 0.05) differences between control conditions (V_half_ = −43.6 ± 4.8 mV; *n* = 13) and application of P4 (V_half_ = 58.1 ± 6.8 mV; *n* = 12) were found for V_half_, the slope of the activation curve was significantly different (control: *k* **=** 21.7** ±** 1.2 mV; P4: *k* = 16.1 ± 1.7 mV; *p* < 0.05; Figure 6D), pointing to a narrower range of voltage‐sensitivity of the channels in presence of the steroid hormone.

#### 2.3.2 Implications for Steroid Hormone Modulation

Steroid hormones, as cholesterol‐derived lipophilic signaling molecules, are well‐suited to modulate ion channel function through direct protein interactions or alterations of membrane properties. Inhibitory effects of steroid hormones on voltage‐gated potassium currents have been reported previously [18, 19]. The inhibitory effects of E2 and P4 on Kv7.2/7.3 currents support the concept that steroid hormones can acutely influence ion channel activity [28, 29, 30]. It has been reported before that cholesterol (10 µM in aqueous solution), a steroid precursor, had a much smaller effect on BK channels than 10 µM corticosterone, a more polar steroid [31]. The strongest effects found in the present study occurred at supraphysiological concentrations, particularly for P4 at 50 µM. Nevertheless, these findings reveal a notable pharmacological sensitivity of Kv7.2/7.3 channels to steroid exposure. The inhibition was more pronounced at depolarized potentials. This effect was accompanied by a steeper activation curve. For E2, effects in the same direction were seen, but did not reach statistical significance. Therefore, future studies have to resolve the exact mechanisms underlying this current reduction. In this respect, the G‐protein‐coupled estrogen receptor 1 (GPER1) may be relevant. It has been shown that effects of steroid hormones at low physiological concentrations (i.e., ≤ 1 µM) require heterologous overexpression of human Kv7.4 channel with GPER1 at the same time [32]. Similar requirements may exist for steroid hormone modulation of Kv7.2/7.3 channels.

In general, our observations should be interpreted within the context of the experimental conditions and model system used. The concentrations used in this study, particularly for steroid hormones, exceed physiological levels. Under the acute application paradigm employed here, higher concentrations are required to ensure sufficient compound availability and interaction with the membrane as compared to long‐term compound incubation during culturing of the cells. Accordingly, this work was not designed to address physiological or clinical relevance in the first place but to provide a fast initial characterization of Kv7.2/7.3 channel modulation in a controlled heterologous expression system. Within this framework, the observed effects reflect pharmacological modulation rather than physiological regulation. Moreover, the concentrations used here were chosen to ensure detectable effects under acute experimental conditions as a screening tool rather than to establish full concentration‐response relationships.

## 3 Conclusions

Our data indicate that CHIM derivatives are suitable tools for modulating Kv7.2/7.3 currents in heterologous expression systems. Nevertheless, their amphiphilic nature suggests that, particularly at higher concentrations, membrane‐mediated effects may contribute to the observed current modulation. Careful control of compound concentration is therefore required to reduce nonspecific membrane effects and to distinguish direct channel modulation from indirect membrane‐mediated mechanisms. The introduction of a fluorescent moiety, as in CHIM‐L‐NBD, markedly expands the functional scope of this scaffold. Beyond pharmacological modulation, such derivatives enable the assessment of membrane localization and may facilitate the analysis of spatial proximity to the Kv7 channel complexes.

The transition from Kv7.2/7.3 current inhibition by CHIM and CHIM‐L to channel activation by CHIM‐L‐NBD highlights how minor structural modifications at the steroidal scaffold can fundamentally alter functional outcome. This switch may arise from altered membrane partitioning, changes in bilayer orientation, or modified interaction with channel‐associated lipid binding sites. Elucidating the molecular basis of this inversion will require systematic structure‐activity studies in combination with electrophysiological measurements and imaging approaches.

In light of recent advances demonstrating that subtle chemical modifications can invert Kv7 channel modulation and enable subtype‐selective targeting, cholesterol‐derived scaffolds such as those studied here may offer a complementary platform for the rational design of future Kv7 modulators. Together, these findings demonstrate differences in the direction and relative magnitude of current modulation induced by structurally related compounds and establish a foundation for future mechanistic and pharmacological studies.

## 4 Experimental

### 4.1 Cell Culture and Transfection

HEK293FT cells were maintained in Dulbecco's Modified Eagle Medium (DMEM) supplemented with 10% fetal bovine serum and 1% penicillin/streptomycin (Pan Biotech).

KCNQ3‐P2A‐KCNQ2‐DsRED: As a first step, cells were plated in 35 mm tissue culture dishes to a 5 × 10^5^ cells/dish density. The day after plating, transfection was performed. In brief, Lipofectamine 3000 reagent and KCNQ2‐P2A‐KCNQ3‐DsRED were diluted in Opti‐MEM medium (Thermo Fisher Scientific). This was followed by mixing the diluted Lipofectamine 3000 reagent and the diluted KCNQ3‐P2A‐KCNQ2‐DsRED (500 ng end concentration) in a 1:1 ratio. It was added to the dishes after incubating the mixture for 12 min at room temperature. Cells were incubated overnight at 37°C with 5% CO_2_ (v/v) in HERACell 150i GP.

KCNQ3‐P2A‐KCNQ2‐GFP: Cells were transfected with 500 ng plasmid DNA per dish using Lipofectamine 3000. For each dish, two solutions were prepared: (A) 50 µL Opti‐MEM containing 500 ng DNA and 0.8 µL P3000 reagent, and (B) 50 µL Opti‐MEM containing 1 µL Lipofectamine 3000. Solutions were incubated separately for 5 min at room temperature, then combined (B into A) and incubated for an additional 20 min before being added dropwise to the cells. After 6 h, the transfection mixture was replaced with fresh culture medium, and cells were analyzed the following day.

#### 4.1.1 Plasmid Design

**Figure 7:**
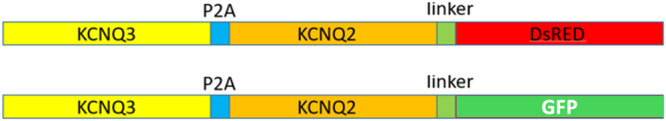
Schematic plasmid card of KCNQ3‐P2A‐KCNQ2‐DsRED/GFP.

Human KCNQ2 (NM_172107.4) was linked via the self‐cleaving P2A sequence GSGATNFSLLKQAGDVEENPGP to human KCNQ3 (NM_004519.4). The KCNQ2C‐terminal end was linked via the flexible linker GGGGSGGGGS to DsRED/GFP (Figure 7). The resulting construct generates proteins KCNQ3 and KCNQ2‐DsRED/GFP in a fixed 1:1 stoichiometry. Transfected cells can be identified by DsRED/GFP fluorescence.

### 4.2 Whole‐Cell Patch‐Clamp Recordings

Recordings were performed at room temperature using the EPC10 USB patch clamp amplifier (HEKA Electronics, Lambrecht, Germany). Patchmaster v2x91 software was used for data acquisition. The protocol was adapted from previous publications [24, 33].

For recordings, the external medium was exchanged for a solution containing (in mmol/L): 140 NaCl, 2 KCl, 10 HEPES, 10 glucose, 1 CaCl_2_, 3 MgCl_2_, and 0.001 tetrodotoxin (TTX). Borosilicate glass pipettes (30‐0066, filamented, Harvard apparatus) were pulled with a HEKA PIP 6 temperature‐controlled pipette puller. Patch pipettes with a resistance of 3–5 MΩ were filled with an internal solution containing (in mmol/L): 10 NaCl, 10 KCl, 85 K‐gluconate, 20 K_3_‐citrate, 10 HEPES, 3 K‐BAPTA, 3 Mg‐ATP, 0.5 Na‐GTP, 15 phosphocreatine, 1 MgCl_2_, and 0.5 CaCl_2_. Cells were held at a potential of −80 mV before stepping to a conditioning potential of −90 mV for 2 s. Thereafter voltage steps to varying test potentials (−80 mV to +40 mV) were applied for 2 s with increments of 10 mV and intervals of 15 s before stepping to a constant potential of −60 mV for 1 s. This protocol was identical across all experiments. Compounds were dissolved in the external solution and applied via a bath perfusion system for 2 min prior to recording to assess acute electrophysiological effects under the standard whole‐cell patch‐clamp conditions, a commonly used approach to evaluate rapid functional effects of compounds under continuous perfusion [34]. For quantification of compound effects, responding and non‐responding cells were taken into account.

Currents were normalized to the peak current density amplitude measured at the end of the 2‐second depolarization step at +40 mV under control conditions for each cell, to account for variability in expression levels and cell size inherent to transient transfection systems and to enable reliable comparison of pharmacological effects across cells. Current‐voltage relationships were obtained by plotting normalized current values as a function of membrane voltage. For better comparison data at +30 mV are provided as relative and absolute current values.

To analyze the time course of the cholesterol (10 µM in chloroform) effect, time‐dependent measurements were performed. For this, a single sweep protocol was used where a step from −90 to +20 mV was repeated every 10 s.

For determining steady‐state current activation, the conductance g was calculated using a liquid junction potential of −14 mV, a reversal potential of −110 mV for K^+^, and the current amplitudes measured at each test potential. Conductance was normalized to the maximal conductance (g/g_max_). By fitting the data points to the following Boltzmann Equation (1) in Origin (OriginPro 2021—version 9.8.0.200), the potential of half‐maximal activation (V_half_ = x_0_) and the slope factor (*k* = dx) of the activation curve were determined.

(1) $$y=\frac{A_{1}-A_{2}}{1+e^{(x-x_{0})/\mathrm{dx}}}+A_{2}$$

Activation and deactivation kinetics were obtained by fitting the current traces to a two‐exponential function using Equation (2) with PyPAS (pyPAS—version 0.9.3) [35].

(2) $$I=I_{0}+A_{1}*e^{-\frac{x-x_{0}}{\tau_{1}}}+A_{2}*e^{-\frac{x-x_{0}}{\tau_{2}}}$$

Statistical differences were determined using a two‐way ANOVA followed by a Tukey's post hoc test with a significance level of 0.05. Outliers were detected using a Grubbs test with a significance level of 0.05. Values were only obtained if the fit converged. Consequently, there are inconsistencies in the indicated sample sizes.

### 4.3 Statistical Analysis

Data are presented as mean ± SEM (*n* = independent cells). Normality was assessed using the Shapiro–Wilk test. Paired Student's *t*‐tests and two‐way ANOVA were applied as appropriate, with *p* < 0.05 considered statistically significant.

### 4.4 Cholesterol Preparation for the Measurements

A stock solution of cholesterol in chloroform or DMSO was prepared and sonicated to ensure adequate solubilization. Prior to the recordings, the stock solution was further diluted in the external recording solution, where no visible precipitation or turbidity was observed. The final solvent concentration did not exceed 0.1% (v/v) and had no detectable effect on Kv7.2/7.3 currents (vehicle control). While DMSO‐based delivery allows acute application, we cannot exclude incomplete membrane incorporation or micro‐aggregation of cholesterol under these conditions. Therefore, the observed effects should be interpreted as pharmacological rather than physiological and with caution regarding effective membrane concentrations.

### 4.5 Synthesis of Cholesterol Analogs

The cholesterol‐based analogs CHIM and CHIM‐L were prepared following an established synthetic route [20]. Briefly, commercially available cholesterol was transformed into 2,3‐cholestandione through a five‐step process. This intermediate was then used to generate the corresponding imidazole derivatives by reaction with either paraformaldehyde or 6‐azidohexanal to generate the corresponding imidazole precursors of CHIM and CHIM‐L, respectively. Final N‐methylation afforded the imidazolium‐containing cholesterol analogs CHIM and CHIM‐L.

CHIM‐L‐NBD was synthesized according to an established protocol [20] via copper‐catalyzed azide‐alkyne cycloaddition (“click” chemistry), in which the azide group of CHIM‐L was coupled to the alkyne‐functionalized fluorescent NBD moiety. CHIM‐L‐NBD is most stable as a dry powder; solutions should be freshly prepared in DMSO and used immediately to avoid NBD dissociation.

### 4.6 Cell Staining and Imaging

For DsRED imaging, cells (~4200 cells/mm^2^) were transfected with 500 ng KCNQ3‐P2A‐KCNQ2‐DsRED using Lipofectamine 3000 (6 h). After 24 h, cells were incubated with 10 µM CHIM‐L‐NBD (5 min, room temperature), fixed with 4% PFA (10 min, room temperature), and mounted in VECTASHIELD HardSet with DAPI. Confocal images were acquired on a Leica TCS SP8 (HC PL APO CS2 20x/0.75 DRY) in sequential mode (pinhole 59.5 µm; no averaging). Contrast was adjusted uniformly.

For the GFP imaging, cells were transfected with 500 ng KCNQ3‐P2A‐KCNQ2‐GFP construct as described for previous experiments. Prior to fixation, cells were incubated with the CellMask membrane stain (Deep Red) in DMEM supplemented with 10% FBS and 1% Penicillin/Streptomycin for 10 min at 37°C. Subsequently, cells were fixed with 4% PFA (10 min, room temperature), washed three times with PBS, and mounted in VECTASHIELD HardSet with DAPI. Confocal images were acquired using a Leica confocal microscope with a 20x objective and a pinhole setting of 1 Airy unit. The line averaging was set to 4.

## Conflicts of Interest

The authors declare no conflicts of interest.

## Data Availability

The data that support the findings of this study are available from the corresponding author upon reasonable request.
